# National Health Insurance Development in China from 2004 to 2011: Coverage versus Benefits

**DOI:** 10.1371/journal.pone.0124995

**Published:** 2015-05-28

**Authors:** Yan Zhang, Wenxi Tang, Xiang Zhang, Yaoguang Zhang, Liang Zhang

**Affiliations:** 1 School of Medicine and Health Management, Tongji Medical College, Huazhong University of Science and Technology, Wuhan, Hubei, 430030, People’s Republic of China; 2 Centre for Health Statistics Information, National Health and Family Planning Commission, People’s Republic of China, Beijing, 100044, People’s Republic of China; National Institute for Viral Disease Control and Prevention, CDC, China, CHINA

## Abstract

**Background:**

The simultaneous improvement of the security capability of China Health Insurance System and its development in the last decade remains uncertain. This study measures the status and trends of reimbursement levels of the China Health Insurance System, as well as to offer policy advice to subsequent insurance reforms.

**Methods:**

The National Reimbursement Ratio was created to determine the reimbursement level of the national health insurance system based on total health expenditure and the covered population. Chinese total health expenditure data from 2004 to 2011 were extracted from China’s Health Statistics according to the standards of the International Classification for Health Accounts by Healthcare Financing.

**Results:**

In 2011, the medical expenditure per capita in China was USD 130.95 and the National Reimbursement Ratio was 26.39%. The National Reimbursement Ratio showed an intense transition from 2004 to 2011, with a sharp decrease from 98.51% in 2004 to 22.44% in 2009, and then a small increase to 26.39% in 2011.

**Conclusion:**

The National Reimbursement Ratio was effective in revealing the reimbursement level of the national health insurance system and in predicting its trends. The challenge to China’s healthcare reform is to switch from increasing insurance coverage to guaranteeing a steady increase in government input and building a powerful supervision mechanism.

## Introduction

The increase in social health insurance coverage in China from less than 15% to more than 95% in 2011 took about 10 years [1]. The China Health Insurance System (CHIS) is a mosaic of different types of mandatory and voluntary insurances because most Chinese residents are covered by either of the three types of basic social health insurance (BSHI), namely, Health Insurance for Urban Employees (HIUE), Health Insurance for Urban Residents (HIUR), and New Rural Cooperative Medical Scheme (NRCMS) [1]. A small proportion of other insurances, such as Free Medical Care, enterprise health insurance, and some commercial insurance schemes, provide supplementary medical insurance to complete the system [2, 3]. The effective combination of these insurance systems has enabled the Chinese government to provide a basic safety net for Chinese residents. Unfortunately, more coverage does not mean more service utilization and better health [4].

According to the *World Health Report 2010*, financial catastrophe and impoverishment fall to negligible levels when direct payments (out-of-pocket, OOP) fall from 15% to 20% of total health expenditure (THE) [5]. In the *Health Financing Strategy for the Asia Pacific Region (2010 to 2015)*, the WHO advocates taking the ratio of OOP to THE as a core indicator to monitor and evaluate the performance of a country or a region in its implementation of a “Universal Coverage” strategy [6]. However, a realistic limitation of this indicator is that as the territorial government expenditure on healthcare occupies a larger proportion of THE in the Health Accounts, enlarging the territorial government expenditure on healthcare could lower the ratio of OOP to THE but it might not reflect a potentially improved financial protection. Thus, reflecting the degree of financial risk sharing by simply comparing OOP to THE is not enough. From a worldwide view, one of the major objectives of most health insurance systems (HIS) is to increase the reimbursement level of patient expenditures, at least in those systems in which patients are paying for services OOP [5,7]. The key social function of health insurance is its ability to determine access to medical care and to share part of the financial risks with patients and their families [8]. Therefore, we suggest that the reimbursement ratio is a more sensitive indicator to assess the security capacity of HIS.

Notably, the policy-defined reimbursement ratio does not take place the actual reimbursement ratio of HIS. All insurances in China have a fee-for-service mechanism through which beneficiaries’ expenditures are reimbursed or compensated later based on the reimbursement lists of drugs, services, and facility standards [4, 9]. Actual reimbursement ratio refers to the ratio of direct payments to hospital expenditures for patients, regardless of the available service range of HIS. In fact, although the policy-defined reimbursement ratio in CHIS has been increasing year by year, the actual reimbursement level has not necessarily increased concurrently.

Comparing the reimbursement levels of different HISs from the perspective of the beneficiaries is impossible because of different benefit lists and cost sharing policies. Insurance benefits are determined by a certain range of policies in a municipality in China. However, the benefit lists and cost sharing policies in different HISs in a municipality are different because of the high development variation of different HISs. For example, the range of coverage drugs of HIUE is 2510, whereas that of NRCMS is 899 in Hubei Province in 2012[10].

The reimbursement levels of HISs among different regions in China are difficult to compare because of the high socioeconomic variation and the composition of HISs. The consumer spending per capita in Nanjing, a sample city in eastern China, is three times higher than in Tibet, a capital city of the western province in 2011 [9]. The main body of HISs in eastern China is HIUE and HIUR, while that in western China is NRCMS. Moreover, the benefit lists of NRCMS in different counties are different.

To sum up, because of the various forms of HISs in China, the actual reimbursement ratios of any HISs do not represent the actual reimbursement ratio of the whole CHIS. To make things worse, many people are covered by more than one HIS. For some rural migrant workers, they are voluntarily covered in NRCMS in their hometown and are usually compulsorily insured again in HIUR in their workplace.

Therefore, we only consider the total reimbursement level. The National Reimbursement Ratio (NRR) can serve as a practical indicator that can truly reflect the whole compensation level of national HIS.

## Materials and Methods

### Methods

First, we designed three new variables, namely, medical expenditure per capita (MEPC), individual payment, and NRR. MEPC is the national total medical expense per capita for all medical services, including expenses for the listed benefits and for any other health services (as part of the health expenditure per capita). These expenses reflect all medical services utilized by all residents. Individual payment is calculated from OOP (taken from THE), which represents the direct disease burden of health services for the individual [11]. The NRR is defined as the ratio of the reimbursement per capita for people covered by all HISs compared with the MEPC for the whole nation, reflecting the average reimbursement level of the entire CHIS. The ratio of the remaining proportion for covered people is the individual payment ratio, which is a new variable compared with NRR, referring to the ratio of OOP per capita to the MEPC for insured people.

Second, dividing the total population into covered population and uncovered population results in two different kinds of OOP, as reflected in the following equation: `

R*Q=Q*γ*C*Ｃ*β＋Q*(１−γ)*Ｃ (I)

R = the OOP per capita by all residents;
Q = the total population;
γ = the national HIS coverage;
C = medical expenditure per capita for all residents;
β = the individual pay ratio under the national HIS.

Thus, we can estimate the individual payment ratio by:

$$\beta =\frac{\frac{\text{R}}{\text{C}}+\gamma -１}{\gamma }$$ (II)

Finally, we could calculate the NRR (α) by:

α=1−β (III)

Here, we have made two assumptions in this calculation, namely, (1) all residents could have an equal medical expenditure; and (2) the reimbursement ratio under different HISs could be substituted by an averaged reimbursement ratio for the national HIS.

Thus, NRR could be obtained from 2 data sources, namely, the total covered population and THE.

### Data Collection

Data about the population covered by different HISs from 2004 to 2011 in China were extracted from the *China Statistical Yearbook of Health and Family Planning 2013* [12]. The Chinese total health expenditure (CTHE) during the same period was taken from the *China National Health Accounts Report 2013* [13].

In 2004, the WHO recommended that all member counties/districts use the International Classification for Health Accounts by Healthcare Financing (ICHA-HF) to measure National Health Accounts by gathering data from funding agencies [11, 14–15]. The new THE classification includes two parts, general government expenditure (HF.1) and private expenditure (HF.2). General government expenditure is made up of expenditures by territorial governments (HF.1.1) and social security funds (HF.1.2). Private expenditure (HF.2) is comprised of private social insurance (HF.2.1), other private insurance (HF.2.2), private household OOP spending (HF.2.3), payments by non-profit institutions (HF.2.4), and payments by corporations (HF.2.5) [14]. The classification of ICHA-HF involves two basic perspectives. First, financing agents, which are the organizations or individuals that directly pay for the health care, that is, third-party-payment arrangements and direct payments by households. Second, primary sources of funding, which bear the ultimate burden of financing [14]. In this kind of analysis, intermediary sources of funding (social security funds, private insurance, and social institutions) are traced back to their origin. All health-related expenditures would be captured in this method. For example, HF.2.3 (OOP) comprise of direct payments, cost-sharing, and co-payments, including all direct payments from residents, regardless if insured or not[16].

After the standard was published, most of member counties/districts reconfigured their National Health Accounts according to the ICHA-HF, but with a few modifications unique to their own situations. For example, Australia removed HF.2.4 because no data was under the item. The Netherlands does not separate HF.2.4 from HF.2.5 because of the small proportion of the total HF.2 [17]. China also reconfigured the Chinese Total Health Expenditure according to ICHA-HF, and renamed China’s Classification for Health Accounts by Healthcare Financing (CCHA-HF) in 2004 [12]. The China National Health Economics Institute, a research institution affiliated with the Ministry of Health, added off-budget expenditure (CHF.1.3) into the general government sector (CHF.1), and divided the private sector (CHF.2) into private insurance (CHF.2.1), private households OOP spending (CHF.2.2), and spending by social institutions (CHF.2.3). However, the CCHA-HF continues to abide by National Health Accounts and System of Health Accounts’ rules [13]. The China National Health Economics Institute collected all data from all health facilities and insurance funds based on CCHA-HF compulsory reporting. All health facilities and insurance funds must submit last year’s data in February annually, including private insurance funds and clinics. Since there were almost no health care suppliers without a license, medical expenses from THE represented the actual medical expenses.

THE calculation in this article adopted the CCHA-HF, and all expenses from the 2004 to 2010 were converted into equivalent expenses in 2011 according to the Healthcare Consumer Price Index in China provided by the *China Statistical Yearbook of Health and Family Planning 2013* [12].

### Data Analysis

Table 1 shows the CTHE in CCHA-HF from 2004 to 2011. The Chinese social security expenditure (CHF.1.2) accounted for all insurance funds, including HIUE, HIUR, NRCMS, and other health insurances [12].

**Table 1:** China’s Total Health Expenditure in CCHA-HF, 2004–2011 (US $, in billions).

	2004	2005	2006	2007	2008	2009	2010	2011
**Total Health Expenditure**	103.69	120.2	140.12	169.23	225.85	274.07	305.17	376.87
**CHF.1 *General government expenditure***	39.38	46.6	56.96	79.42	112.81	143.88	165.74	210.4
**CHF.1.1 *Territorial government expenditure***	14.31	17.49	19.93	23.8	31.53	44.28	52.27	63.14
**CHF.1.2 *Social security expenditure***	21.72	25.23	32.64	50.09	75.28	93.08	106.35	141.65
**CHF.1.3 *Off-budget expenditure***	3.35	3.88	4.4	5.52	6.01	6.52	7.12	5.6
**CHF.2 *Private expenditure***	64.32	73.61	83.16	89.81	113.04	130.19	139.43	166.48
**CHF.2.1 *Private insurance***	3.51	4.26	5.37	5.61	9.1	8.97	10.35	10.71
**CHF.2.2 *Private household OOP spending***	55.62	62.75	69.09	74.55	91.3	102.66	107.7	131.04
**CHF.2.3 *Social institutions spending***	5.19	6.59	8.7	9.64	12.64	18.55	21.38	24.73

Source of data: National Health and Family Planning Commission of China: *China National Health Accounts Report 2013*. Beijing: 2013.

We took private household OOP spending (CHF.2.2) as the total OOP expenditure. Total medical expenditure could then be calculated from social security expenditure (CHF.1.2) with the exclusion of any unspent BSHI funds, private insurance (CHF.2.1), and private household OOP spending (CHF.2.2). The total social security expenditure (CHF.1.2) without unspent BSHI funds and private insurance (CHF.2.1) represented the entire reimbursement fee of CHIS.

## Results

### China Basically Realized Universal Insurance Coverage in 2011

By the end of December 2011, the population covered by the NRCMS was 832 million and by HIUE and HIUR together was around 473 million. The coverage of the entire BSHI reached 96.86% of the total population (Table 2). Nearly 8.2 million civil servants were covered by Free Medical Care and another 20–25 million people were covered by private health insurance before 2009. After the 2009 policy reform, most civil servants were mandatorily turned over to HIUE, and an unknown number of people with private health insurance were moved voluntarily into HIUR. Thus, only an estimation of the change in supplementary insurance coverage exists. The total population covered by supplementary medical insurance was 2% of the total population before 2009 and 20% of the uncovered population after 2009. Thus, we can conclude that over 95% of the Chinese population was insured by CHIS, which we basically considered to be universal insurance coverage.

**Table 2:** The population coverage by CHIS, China 2004–2011 (in millions).

	2004	2005	2006	2007	2008	2009	2010	2011
**The country’s population**	1299.88	1307.56	1314.48	1321.29	1328.02	1334.50	1340.91	1347.35
**HIUE**	124.04	137.83	157.32	180.20	199.96	219.37	237.35	252.27
**HIUR**	—	—	—	42.91	118.26	182.10	195.28	221.16
**NRCMS**	80.79	179.06	412.65	726.24	815.18	833.09	835.60	831.63
**BSHI coverage (%)**	15.76	24.24	43.36	71.85	85.34	92.51	94.58	96.86
**CHIS coverage (%)**	17.76	26.24	45.36	73.85	87.34	94.01	95.66	97.49

Source of data: National Health and Family Planning Commission of China: *China Statistics Yearbook of Health and Family Planning 2013*. Beijing: 2013.

Note: BSHI consists of HIUE, HIUR and NRCMS in China.

### Reimbursement Level of the CHIS Experienced a Major Challenge

**Fig 1:**
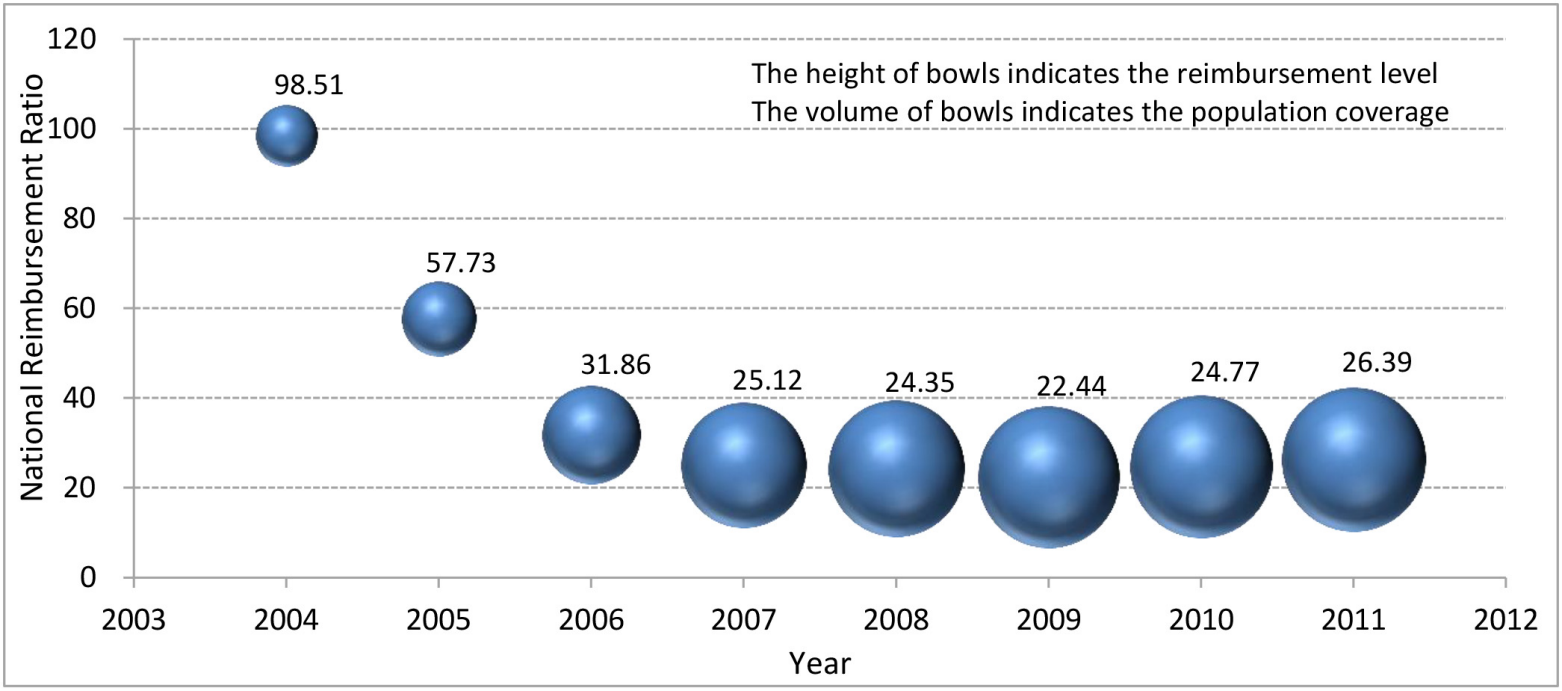
Trends in the National Reimbursement Ratio in China, 2004–2011. The height of bowls indicates the reimbursement level of the China Health Insurance System, and the volume of bowls indicates the population coverage under the China Health Insurance System.

We identified the unspent BSHI funds from 2004 to 2011 (Table 3), and OOP payments by Chinese residents and reimbursements by CHIS were calculated from 2004 to 2011 (Table 4). Noticeably, the other unspent HISs funds were not taken into consideration due to data absence and a little proportion. Individual pay ratio and NRR were then provided (Table 5). We also depicted the historical trends in NRR change and the increasing expansion of CHIS coverage (Fig 1).

**Table 3:** Unspent BSHI funds, 2004–2011 (US$, in billions).

	2004	2005	2006	2007	2008	2009	2010	2011
**HIUE**	13.09	17.74	24.95	35.69	53.37	66.80	72.41	92.34
**HIUR**	—	—	—	0.43	1.66	2.74	4.08	5.71
**NRCMS**	0.35	0.55	1.38	2.61	4.68	5.04	6.77	8.93
**BSHI**	13.44	18.29	26.33	38.73	59.71	74.59	83.26	106.98

Source of data: National Health and Family Planning Commission of China: *China Statistical Yearbook of Health and Family Planning 2013*. Beijing: 2013.

**Table 4:** Health expenditure and reimbursement, total and per capita, China 2004–2011 (in US $).

	2004	2005	2006	2007	2008	2009	2010	2011	Annual growth 2004–2009 (%)	Annual growth 2009–2011 (%)	Annual growth 2004–2011 (%)
**Total medical expenditure (billion)^①^**	67.41	73.95	80.76	91.53	115.96	130.12	141.14	176.42	14.06	16.44	14.73
**Medical Expenditure per capita**	51.86	56.56	61.44	69.27	87.32	97.49	105.25	130.95	13.45	15.9	14.15
**Total reimbursement (billion) ^②^**	11.79	11.2	11.67	16.98	24.66	27.45	33.44	45.38	18.41	28.57	21.23
**Total OOP (billion) ^③^**	55.62	62.75	69.09	74.55	91.3	102.66	107.7	131.04	13.04	12.98	13.02
**OOP per capita**	42.79	47.99	52.56	56.42	68.75	76.92	80.32	97.26	12.45	12.45	12.45

Note:

①Total medical expenditure = CHF. 1.2 + CHF. 2.1 + CHF. 2.2(Table 1) – Unspent BSHI funds (Table 3)

②Total reimbursement = CHF. 1.2 + CHF. 2.1(Table 1) – Unspent BSHI funds (Table 3)

③Total OOP = CHF. 2.2(Table 1).

**Table 5:** National Reimbursement Ratios, China 2004–2011 (%).

	2004	2005	2006	2007	2008	2009	2010	2011
**Insurance Coverage**	17.76	26.24	45.36	73.85	87.34	94.01	95.66	97.49
**Individual pay ratio^①^**	1.49	42.27	68.14	74.88	75.65	77.56	75.23	73.61
**National Reimbursement Ratio^②^**	98.51	57.73	31.86	25.12	24.35	22.44	24.77	26.39

Note:

①Individual pay ratio = ((OOP per capita)/MEPC + Insurance coverage – 1) / Insurance coverage. See Table 4 for MEPC

②National Reimbursement Ratio = 1 – Individual pay ratio.

In 2011, the CHIS coverage in China was about 97.5%, and the MEPC was up to USD 130.95. The NRR was only about 26.4%. For the 1.31 billion covered people, OOP accounted for 73.61% (USD 96.4) of the patient burden. Moreover, a transition in the change of NRR from 2004 to 2011 existed. NRR experienced a sharp decrease from 98.51% in 2004 to 22.44% in 2009, and then slightly climbed to 26.39% in 2011.

## Discussion

### Change of National Reimbursement Ratio indicates change of CHIS

The interpretation of our results should be considered from a historical perspective. During the period of study, CHIS made great changes in its entire structure. In 2004, when CHIS was at an early stage, HIUE had been fully established while NRCMS was just starting. The dominant insurance schemes in CHIS were only HIUE and Free Medical Care. The beneficiaries of both insurance schemes were mostly people with income levels above the average level. These groups were privileged to have access to a higher level of medical service, and thus, remarkably higher medical expenditure, so that the absolute reimbursement figures were correspondingly much higher at that time. As a result, the calculated NRR figure reached 98.51% in 2004, which means that the reimbursement per capita under CHIS was nearly equal to the MEPC in China and adversely indicated an unequal access to medical care by covered and uncovered groups.

With the rapid development of CHIS, the NRCMS was established within five years and soon covered more than 800 million rural Chinese people. While the covered population sustained an expanding growth rate of 28.20% from 2004 to 2011, the NRR experienced a decrease from 98.51% to 26.39% in the same period. Upon closer observation, the NRR decrease occurred in two different stages, with a sharp decline from 2004 to 2009 and then a small rebound after 2009. Telling why 2009 became a turning point is not hard. From 2004 to 2009, the annual growth rate of the covered population was 40.29%. However, since 2009 when the population coverage under BSHI reached nearly 95%, the expansion slowed down to a moderate growth rate of 2.32% since then. With the growth rates of reimbursement and MECP having both maintained steady levels before and after 2009, the performance of NRR varied mainly with enrollment.

### China Health Reform should Focus on the Government Input and Supervision Construction

Looking back, the shrinking NRR from 2004 to 2009 indicated that although China achieved great success in expanding coverage, the OOP per capita was increasing at the same time with an annual growth rate of 12.45%. We should always keep in mind that people with insurance coverage might not mean that they are automatically insured in health, and would not guarantee that insured people can get healthcare they should deserve [4]. However, the annual growth rate of OOP compared with MEPC shows a significant decreasing trend (12.45% vs 14.15%), and the situation is the same when comparing growth in expenditure on household consumption per capita (18.02%) [11]. We can therefore conclude that the CHIS improved access to medical services, and the capacity for risk sharing is one of the significant success of CHIS from the perspective of society as a whole.

Under publicly financed system, more insured people will inevitably result in lower security level of the insured population, with the reimbursement level mainly determined by the input from public finance [5]. Public finance input is under the control of the government, and the main body of CHIS financing comes from government subsidy (especially NRCMS and HIUR). A huge part of the population was enrolled, but the increase in total reimbursement through public finances was still greater than the increase in MEPC from 2004 to 2011 (21.23% vs 14.15%). Thus, NRR was revived to 26.39%.We can thus make a prediction that from 2012 onwards, the proportion of CHIS coverage will not change tremendously, as the MEPC is likely to increase year by year under the influence of the combined effect of growing patient demands, medical technical advances, and social development. As long as the growth rate of total reimbursement remains steadily higher than that of MEPC, then the NRR of CHIS will continue to increase. In fact, after a year, the growth rates for total reimbursement and MEPC were about 23.39% and 17.61% in 2012, respectively, and the NRR reached about 28.88%, proving our prediction. Thus, given the fixed population coverage and increasing MEPC, the future trend of NRR will merely depend on government input. Compared with the latest health statistics from the Organization for Economic Co-operation and Development (OECD) and other data sources, the proportion of HF.1 (55.5%) in China was lower than those in Canada (66.4%), Australia (64.4%), and Japan (81.4%) in 2011, but similar to that of Taiwan in 2005 (53%)[18], even if the public input increased greatly in recent years.

A gap exists when NRR is compared with the official average reimbursement rates for listed services in 2011. NRR was 26.39%, and the latter was documented to be between 50% to 60%, which might indicate that the benefit lists of available services under CHIS were too narrow to meet the residents’ demands, both reasonable and luxurious [19]. Consistent with other research, we found that inappropriate admissions and luxurious healthcare consumption have been sharply increasing year by year and are important driving factors to the increase of MEPC [20,21]. For example, the caesarean section rate increased from 19.2% in 2003 to 36.3% in 2011 [22]. To raise the reimbursement level of CHIS, the Chinese government should focus not only on more input, but also on insurance performance supervision.

### National Reimbursement Ratio Calculation by Means of THE

THE is a common, important, and practical tool used worldwide, and is recipient- and user-friendly. The general concept for calculating NRR is to statistically analyze medical expenditure and OOP by means of THE, and then determine the NRR from the equation based on the given proportion of the covered population.

According to the National Health Services Survey in China, catastrophic health expenditure rate for all households decreased from 14.0% in 2008 to 12.9% in 2011[22]. During the same period, the ratio of OOP to THE decreased from 40.4% to 34.8% and NRR increased from 24.35% to 26.39%. Thus, we can say that NRR can more sensitively reflect the relationship of reimbursement to medical expenditure than the ratio of OOP to THE because it directly reflects the level of cost-sharing of the national HIS and ignores individual differences.

This method has three explanations. First, the core equation is based on two aforementioned assumptions. However, real world situation cannot fully satisfy these assumptions. For example, the HIUE benefits are maintained as before, and are far higher than HIUR and NRCMS over the past 10 years because HUIE has a high policy-defined reimbursement ratio and a wide available services range. These assumptions can help establish an equation that reflects the relationship between variables from available sources, as well as to make it comparable among NRRs in different years that are less influenced by the scheme differences in a year. We therefore suggest NRR as a useful tool for reimbursement level analysis similar to the average expenditure on household consumption per capita. Second, with regard to the heterogeneity in different insurance systems within CHIS, wide gaps exist in the CHIS in the aspects of fund-raising, reimbursement level, and benefit lists. Therefore, we focused on the whole reimbursement trends rather than the differences between insurance systems. Third, this method reflects the reimbursement level for total medical expenditure in the whole country, which slightly conflicts with the fundamental concept of BHIS to promote access to basic healthcare, but not all healthcare.

## Conclusions

In conclusion, the implementation of CHIS has been proven to be a success because: (1) the coverage improved to nearly 100% and (2) the disease burdens of residents have been slightly alleviated. In the past decade, the reimbursement level has been relatively low compared with fast-growing medical expenditure, and thus, seemed to have a limited effect on improving the level of security of insured people. Our conclusion should remind the government that multiple tasks still need to be completed, including raising the entire level of benefits for the insured population and preventing them from falling into poverty in case of severe sickness. Strategies for further development of CHIS must emphasize on raising the reimbursement level and extending benefits for suffering individuals. To achieve this goal, the government should maintain a steady increase of public finance or think of other financial resources to better share the risks and put more effort into building a correspondingly powerful supervision mechanism.
